# Screening Allelochemical-Resistant Species of the Alien Invasive *Mikania micrantha* for Restoration in South China

**DOI:** 10.1371/journal.pone.0132967

**Published:** 2015-07-15

**Authors:** Ai-Ping Wu, Zi-Li Li, Fei-Fei He, Yan-Hong Wang, Ming Dong

**Affiliations:** 1 Ecology Department, College of Bioscience & Biotechnology, Institute of Ecology, Hunan Agricultural University, Changsha, 410128, China; 2 School of Agriculture, Yunnan University, Kunming 650091, China; 3 School of Forestry and Bio-technology, Zhejiang Agriculture & Forestry University, Hangzhou, 311300, China; 4 College of Life and Environmental Sciences, Hangzhou Normal University, Hangzhou, 310036, China; Agroecological Institute, CHINA

## Abstract

To screen allelochemical-resistant species of the alien invasive weed *Mikania micrantha*, we studied the allelopathic inhibition effects of the leaf aqueous extract (LAE) of *Mikania* on seed germination and seedling growth of the 26 species native or naturalized in the invaded region in South China. Seed germination was more strongly negatively affected by LAE than seedling growth. Responses of seed germination and seed growth to LAE differed differently among the target species. LAE more strongly negatively affected seed germination, but less strongly negatively affected seedling growth, in non-legume species than in legume species. LAE more strongly negatively affected seed germination and seedling growth in native species than naturalized exotic species. Therefore, naturalized exotic non-legume seedlings are more suitable than seeds of native legume species for restoration of *Mikania*-invaded habitats.

## Introduction

Many introduced exotic plant species become invasive in the new habitats [1–3]. Invasive plant brings significant damage to forests, farmlands, and orchards; and results in great loss of native species diversity, significantly reduces stabilities of microbial communities and food webs; and even alters mineral cycling, so plant invasion can greatly damage native plant communities and cause tremendous ecological and economical problems [4–7]. As a component of global change, plant invasion is considered as the second greatest threat to global biodiversity [8–9], for an example, kudzu (*Pueraria lobata*) invasion increases emissions of nitric oxide and ozone pollution [10].

Various methods have been developed to control invasive plants in order to restore native plant communities [11–13]. However, restoration in habitats invaded by some invasive plant species such as *Mikania micrantha* is sometimes difficult even though the invasive plants have been controlled or removed. These invasive plants have the ability to affect soil quality through the release of natural plant toxins known as allelochemicals that make these plants good invaders. These allelochemicals may persist in the soil for a long period of time and thus greatly limit the establishment of target communities [14–17] Accordingly, selecting allelochemical-resistant species is an important step in restoration of invaded communities [15], so far only a few studies have been conducted to screen allelochemical-resistant species for restoration[15, 18–19].

In previous studies, only native species were considered as the target species for restoration and thus only native species were screened for allelochemical-resistance [15, 20–21], this was because restoration with these species is considered to be safe [15]. However, in most regions of the world, many introduced species with long history have been naturalized and considered as an essential part of the ecosystems [22]. As a result, these naturalized, exotic species should be included in allelochemical-resistance screening program for community restoration.

Legumes can use free N_2_ by nitrogen fixation directly as an additional nitrogen source through symbiotic root bacteria and have the competitive advantages over non-legumes especially when nitrogen availability is poor [23]. While, the advantages of legumes are not important when both legumes and non-legumes are under allelopathic conditions because allelochemicals can directly and indirectly affect nodulation formation and nitrogen fixation in legume species [24–26], which greatly decreases the nitrogen availability of legume species rather than non-legume species.

In South China, large areas of agricultural and natural lands have been severely invaded by *Mikania micrantha*, a perennial vine weed native to South America[27–28]. *Mikania* causes significant damage to forests, farmlands and orchards, alter physical and chemical properties of soil, affect nutrient cycling; and change or decline plant, animal and microbe diversity [29–31]. Many parts of *Mikania*, residue even soil beneath the plant stand have been proved allelopathic to greatly inhibit seed germination and seedling growth of some species [32–33].

We collected 26 different species in ecosystem invaded by *mikania* to screen their allelochemical resistance for restoring the *Mikania*-degenerated communities in South China. These species consists of legumes and non-legumes and natives and naturalized non-natives. We expected that legume species were less resistant to allelochemicals than non-legume species because *Mikania* could affect soil microbial community and nitrogen cycling of the ecosystems [34–36]. Furthermore, we hypothesized that there was no significant different allelochemical resistance between exotic and native species due to their long history of co-evolution.

## Materials and Methods

### Leaf extracts of *Mikania*

About 10 kg of *Mikania* fresh leaves were collected from Shengzhen (114° 04′ E, 22° 37′ N, 62m asl.) in July 2012. We state clearly that no specific permissions were required and the field studies did not involve any endangered or protected species. Morever, we had no vertebrate studies in this research. Leaves were dried at 40°C for 72h, dry leaves were processed into fine powder by a milling machine and passed through a 0.45 μm mesh. All of the fine powder was saturated with due distilled water in glass pots and agitated with a glass stick at room temperature (25±3°C) to get 5% (m/v) concentration of aqueous extract. This solution was passed through two layers of filter paper to remove solid materials after extracting for 24 h. Three concentrations of aqueous extract were set in this study: full strength (5%), decimus strength (0.5%), and centesimal strength (0.05%), and ranked high concentration, intermediate concentration and low concentration, respectively. In allelopathic experiments, 5% (m/v) extracts are commonly used and this concentration is considered higher than nature [2, 37], so two lower concentrations (0.5% and 0.05%) were set in this experiment. The pH of the aqueous extracts fluctuated from 6.0 to 7.0. All of the aqueous extracts were kept at 4°C until use.

### Seed sources of target species

Seeds of the 26 species (see Table 1) were purchased from China National Tree Seed Corporation and Chinese Academy of Agricultural Sciences, which were collected in South China in 2011 and 2012 when seeds were matured and then refrigerated at 4°C. The seeds were surface sterilized with 5% peroxide hydrogen for 20 minutes to exclude other inhibition effects such as toxins from microorganisms, then rinsed with enough distilled water. To get the seed size of every species, 100 (large) or 1000 (small) seeds were weighted minimum to 0.01g and replicated three times.

**Table 1:** Name and traits of the 26 target species.

ID	Family	Scientific name	Native	Legume	Seed size^1^
1	Amaranthaceae	*Amaranthus tricolor*	Yes	No	0.68±0.12
2	Casuarinaceae	*Casuarina equisetifolia*	No	No	1.25±0.23
3	Compositae	*Chrysanthemum coronarium*	Yes	No	1.85±0.36
4	Compositae	*Lactuca sativa*	Yes	No	1.22±0.21
5	Compositae	*Lactuca sativa var*.*ramosa*	Yes	No	1.05±0.18
6	Convolvulaceae	*Dichondra repens*	Yes	No	1.53±0.42
7	Cruciferae	*Raphanus sativus*	Yes	No	7.54±1.63
8	Cucurbitaceae	*Cucumis sativus*	Yes	No	32.16±5.48
9	Cucurbitaceae	*Cucurbita moschata*	No	No	225.68±23.51
10	Cucurbitaceae	*Cucurbita pepo*	No	No	175.36±16.25
11	Gramineae	*Lolium perenne*	No	No	1.92±0.34
12	Gramineae	*Poa acroleuca*	Yes	No	0.37±0.06
13	Hamamelidaceae	*Liquidambar formosana*	Yes	No	4.42±1.02
14	Leguminosae	*Acacia dealbata*	No	Yes	12.52 ±1.63
15	Leguminosae	*Albizia julibrissin*	Yes	Yes	40.06±2.86
16	Leguminosae	*Amorpha fruticosa*	No	Yes	10.51±1.03
17	Leguminosae	*Gleditsia sinensis*	Yes	Yes	475.32±29.36
18	Leguminosae	*Gymnocladus chinensis*	Yes	Yes	254.40±31.03
19	Leguminosae	*Medicago sativa*	Yes	Yes	1.75±0.68
20	Leguminosae	*Robinia pseudoacacia*	No	Yes	20.90±3.21
21	Leguminosae	*Trifolium repens*	No	Yes	0.62±0.08
22	Lythraceae	*Lagerstroemia indica*	Yes	No	2.65±0.29
23	Polygonaceae	*Rumex aquaticus*	Yes	No	4.25±1.27
24	Solanaceae	*Capsicum annuum*	No	No	5.50±1.39
25	Solanaceae	*Lycopersicon esculentum*	No	No	3.05±0.83
26	Taxodiaceae	*Cunninghamia lanceolata*	Yes	No	7.41±1.86

^1^Mean ± SE (g 1000^−1^ seeds), N = 3

### Seed germination experiment

Four concentrations of extracts were used in this experiment: control (distilled water), low concentration (0.05%), intermediate concentration (0.5%), and high concentration (0.5%).In each treatment, 50 (small) or 30 (large) seeds of each species were placed in a separate Petri dish lined with 9-cm (20-cm for *Cucurbita moschata*, *Cucurbita pepo*,*Gleditsia sinensis* and *Gymnocladus chinensis*) of two pieces of filter paper, and 5 ml (40 ml for *Cucurbita moschata*, *Cucurbita pepo*, *Gleditsia sinensis* and *Gymnocladus chinensis*) of due extract were added in. There were 6 replicates per treatment per species. The covered Petri dishes of all species were then incubated in 6 culture boxes of 14 h photoperiod at 25°C and more than 75% relative humidity for germination. Seeds were considered to be germinated when the radicle length was over 2 mm and germination was recorded every 24 h. The day when the first seed germinated in each dish was considered as the initial germination time of the replicate. Germination rate (GR) was calculated according to Saxena et al. [38] as GR = (N_1_×1) + (N_2_-N_1_) ×1/2+ (N_3_-N_2_) ×1/3+…+ (N_n_-N_n-1_) ×1/n, where N_n_ is the number of germinated seeds obtained at the first (1), second (2), third (3), …, (n-1), and (n) days. The experiment was terminated when no seeds germinated lasting for three consecutive days for each species and seedlings of the controls were cultured until the cotyledons were totally open for the following seedling growth experiment. Raw data of germination percentage (GP), initial germination time (IGT) and germination rate (GR) were converted to a percentage of the control.

### Seedling growth experiment

The experiment was conducted from August to October 2012, in green-house Changsha, Hunan Province, China, the temperature ranged from 25°C to 39°C. We selected 24 strong and similar individual seedlings of each species to conduct this experiment. Each individual seedling was transplanted to a rectangular plastic pot with 120 cm^2^ soil surface and pre-weighted 0.8 kg of quartz sand inside. In this experiment we used four concentrations of extracts as seed germination experiment: control (distilled water), low concentration (0.05%), intermediate concentration (0.5%), and high concentration (0.5%), resulting a total 104 treatments (4 concentrations of extracts × 26 target species = 104 treatments), and each treatment replicated 6 times. Four days after transplanting, 10 ml standard Hoagland culture solution was added to each pot for supply nutrient for the seedling growth and supplied the same amount of nutrient weekly after that (four times in all). Similarly, three days after supplying nutrient, 15 ml due aqueous extract was added to each pot avoiding any leaching of the liquid solutions and watered extracts every week. The control was added the same amount of tap water on the day when applying nutrient or extracts. In the first two weeks of the experiment, 30 ml tap water was added to each pot every day (excluding the days supplying nutrient and adding extracts) and changed to 40 ml later. The highest concentration of extract (5% or 50 g L^-1^) watered four times each pot (3 g month^-1^) was calculated to be similar to leachates produced by a field total biomass of *Mikania* (10 t ha^-1^)three times higher in view of fluctuant biomass in a high productive year [39–40]. We set another two lower concentrations (0.5% and 0.05%) because not all of the biomass was total leached in such a short time (4 weeks) under field condition.

Seedlings were harvested one week later after adding extracts for the fourth time. Roots were separated from sand by soaking the pot in water for 10 minutes and softly washing the sand away. Chlorophyll fluorescence was measured using achlorophyll fluorometer (PAM-101, Walz, Effeltrich, Germany). Initial fluorescence (F_0_) was recorded on the first euphylla (the fifth lobule for compound leaf) of every species, adapted to darkness for more than 30 minutes. The maximum chlorophyll fluorescence (Fm) was obtained after a single saturating radiation pulse was applied. The maximum efficiency of PS2 photochemistry, namely Fv/Fm was calculated according to Demmig-Adams et al. [41]. All leaves of every seedling were scanned by a scanner (Epson Perfection 4870 Photo) and the whole leaf area of the seedling was analyzed by an analysis software (WinFOLIA 2004a, Regent Instruments Inc., Qúebec, Canadamachine), then the plant was separated into root and shoot. Root and shoot dry weight was measured after drying at 80°C for 72 h, respectively. Leaf area (LA), Fv/Fm, root dry weight (RW) and shoot dry weight (SW) were all expressed as a percentage of the control.

### Data analysis

Two-way analysis of variance (ANOVA) was used to test the effect of species identity and leaf extract of *Mikania* on seed germination and seedling growth (SPSS 13.0, SPSS Inc., Chicago). Homogeneity of variances was tested by using Levene’s test and the least significant differences (LSD) between means of the same species and the 26 species were determined at P < 0.05, and the differences between means (three concentrations excluding control) of legume VS non-legume species and native VS exotic species were estimated at 95% confidence level.

## Results

### Seed germination

**Fig 1:**
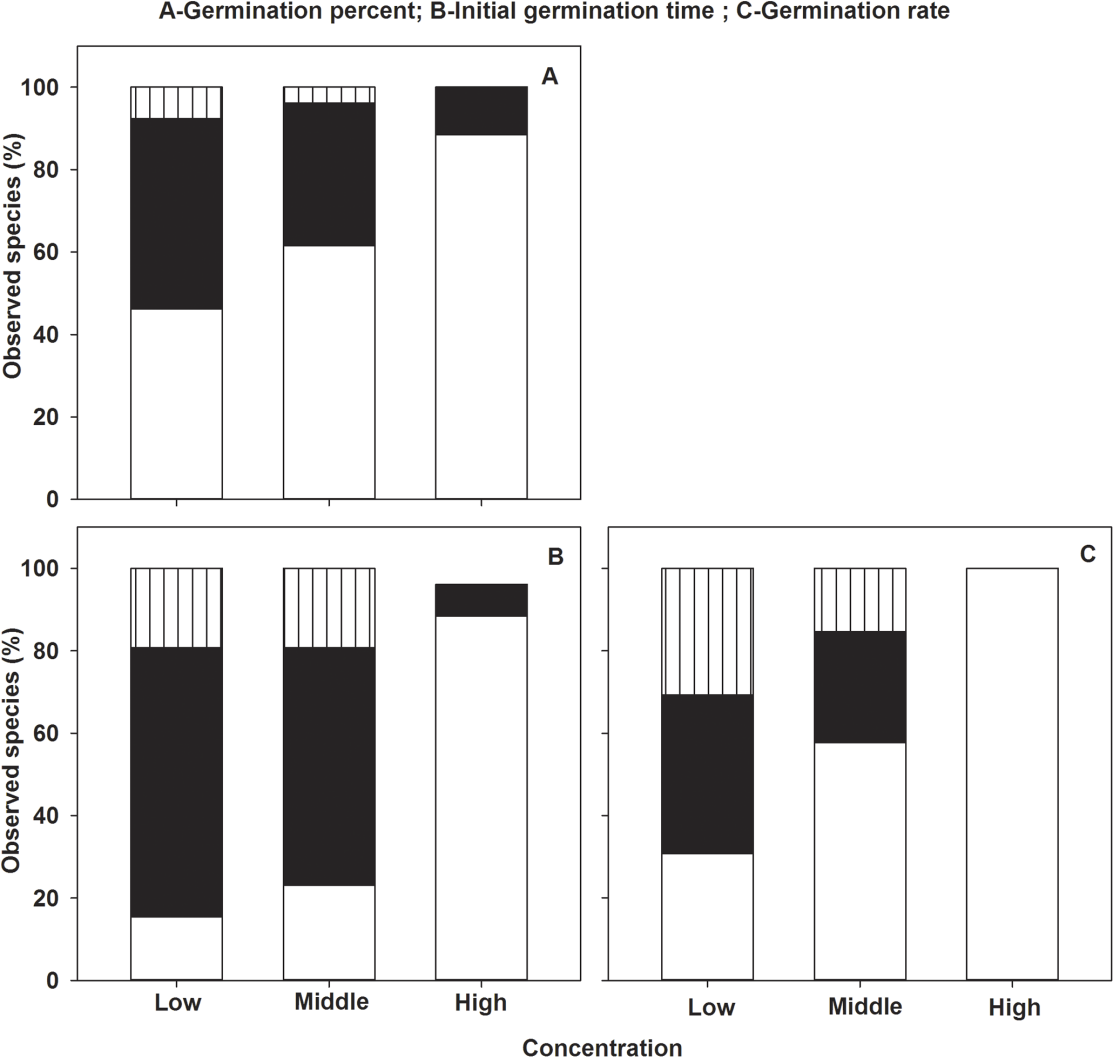
The three different allelopathic effects of *Mikania* on seed germination percent (A), initial germination time (B) and germination rate (C) of the 26 target species in low, middle and high concentrations of leaf aqueous extract; negative effect (white), neutral effect (black) and stimulated effect (batched).

**Fig 2:**
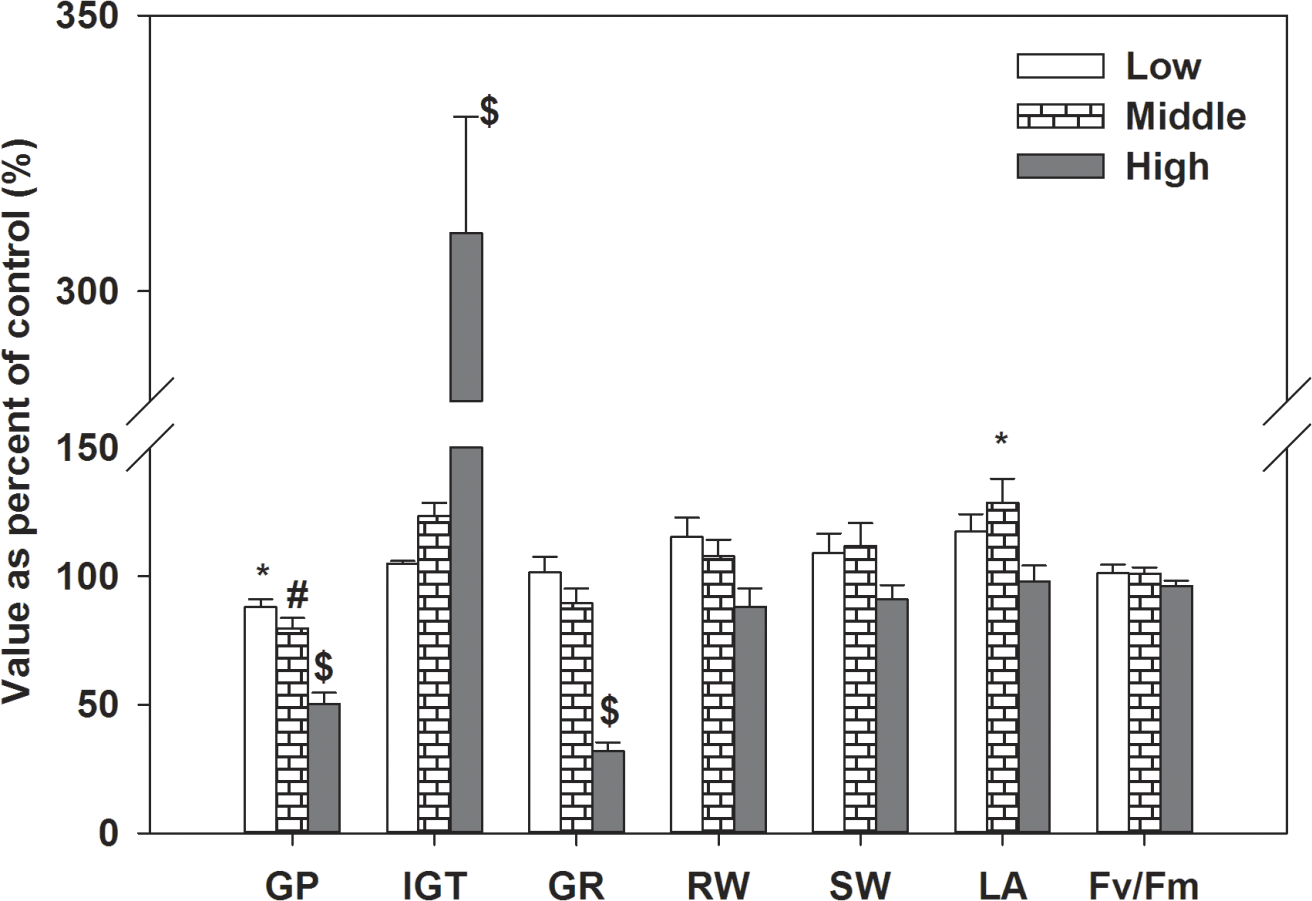
The mean allelopathic effects of Mikania on seed germination percent (GP), initial germination time (IGT), germination rate (GR), seedlings root dry weight (RW), shoot dry weight (SW), leaf area (LA) and Fv/Fm of the 26 target species; bars represent standard errors;“#”: p<0.01; “$”: p<0.001.

**Fig 3:**
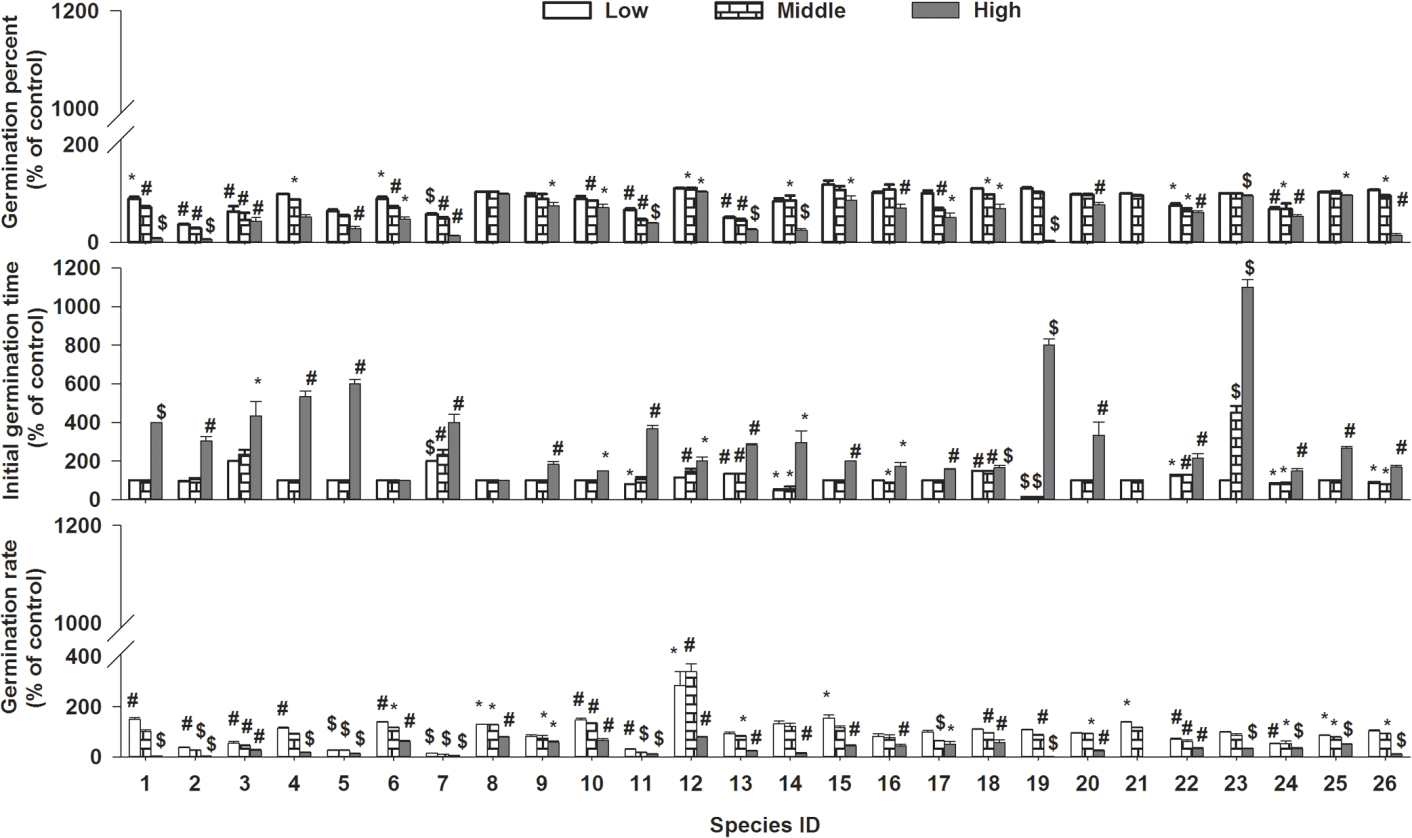
The allelopathic effects of Mikania on seed germination percent, initial germination time and germination rate of the 26 target species at three concentrations; bars represent standard errors;“*”: p<0.05; “#”: p<0.01; “$”: p<0.001.

The *Mikania* leaf extract significantly affected seed germination percentage, initial germination time and germination rate of the 26 species and the inhibition strength increased with extract concentration (Figs 1–3, ANOVA, p<0.01, Table 2). The three measured indices were not significantly affected and even promoted in low and middle concentration for a few species, while most species (88.46%-100%) were significantly affected in high concentration, and germination of *Trifolium repens*was totally suppressed (Figs 1 and 3). Accordingly, the percent of species which were negative affected was increased with increasing concentration (Fig 1). The responses to allelopathic extract were different among the 26 bioassay species and significant two-way interactions between species and concentration were detected for all measured parameters (Fig 3, ANOVA, p<0.01, Table 2)

**Table 2:** Effects of leaf extract concentrations of *Mikania*, species identity and the interaction on seed germination and seedling growth.

	Seed germination		Seedling growth	
Source	F	*P*	F	*P*
	Germination percent		Root dry weight	
Concentration (C)	37.87	<0.001	11.61	<0.001
Species (S)	3.52	<0.001	2.30	0.003
C × S	13.12	<0.001	9.24	<0.001
	Initial germination time		Shoot dry weight	
Concentration (C)	21.84	<0.001	16.82	<0.001
Species (S)	2.08	0.007	1.82	0.023
C × S	39.01	<0.001	2.81	<0.001
	Germination rate		Leaf area	
Concentration (C)	35.05	<0.001	3.79	0.014
Species (S)	3.89	<0.001	3.45	<0.001
C × S	16.72	<0.001	2.81	<0.001
			Fv/Fm	
Concentration (C)			3.24	0.027
Species (S)			2.68	0.001
C × S			2.02	<0.001

F and *P* of ANOVA are given; df (3, 620) is for the concentration effect, (25, 598) for the species effect and (75, 548) for the interaction effect.

### Seedling growth

**Fig 4:**
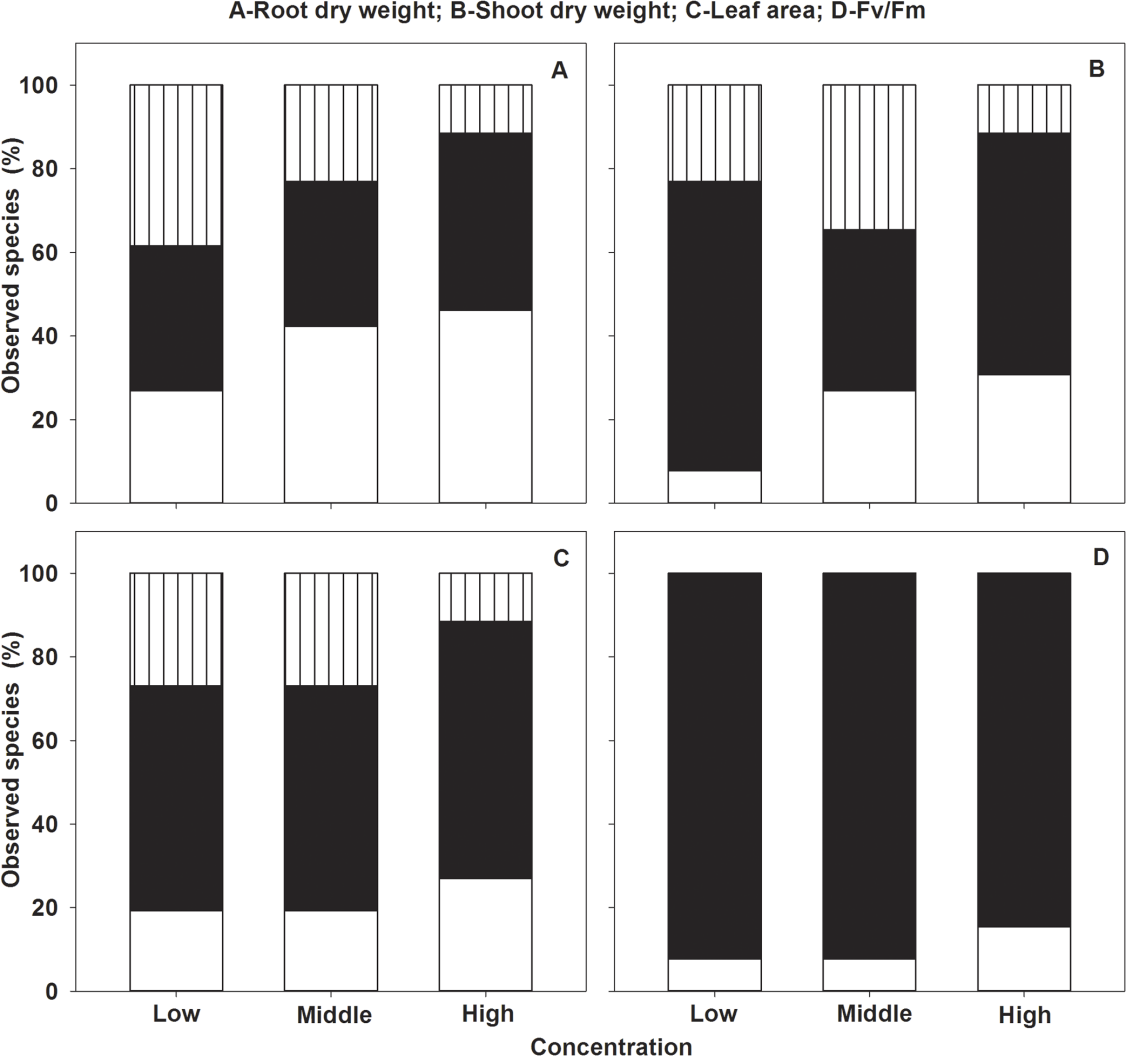
The three different allelopathic effects of Mikania on seedlings root dry weight (A), shoot dry weight (B), leaf area (C) and Fv/Fm (D) of the 26 target species in low, middle and high concentrations of leaf aqueous extract;negative effect(white), neutraleffect (black) and stimulated effect (batched).

**Fig 5:**
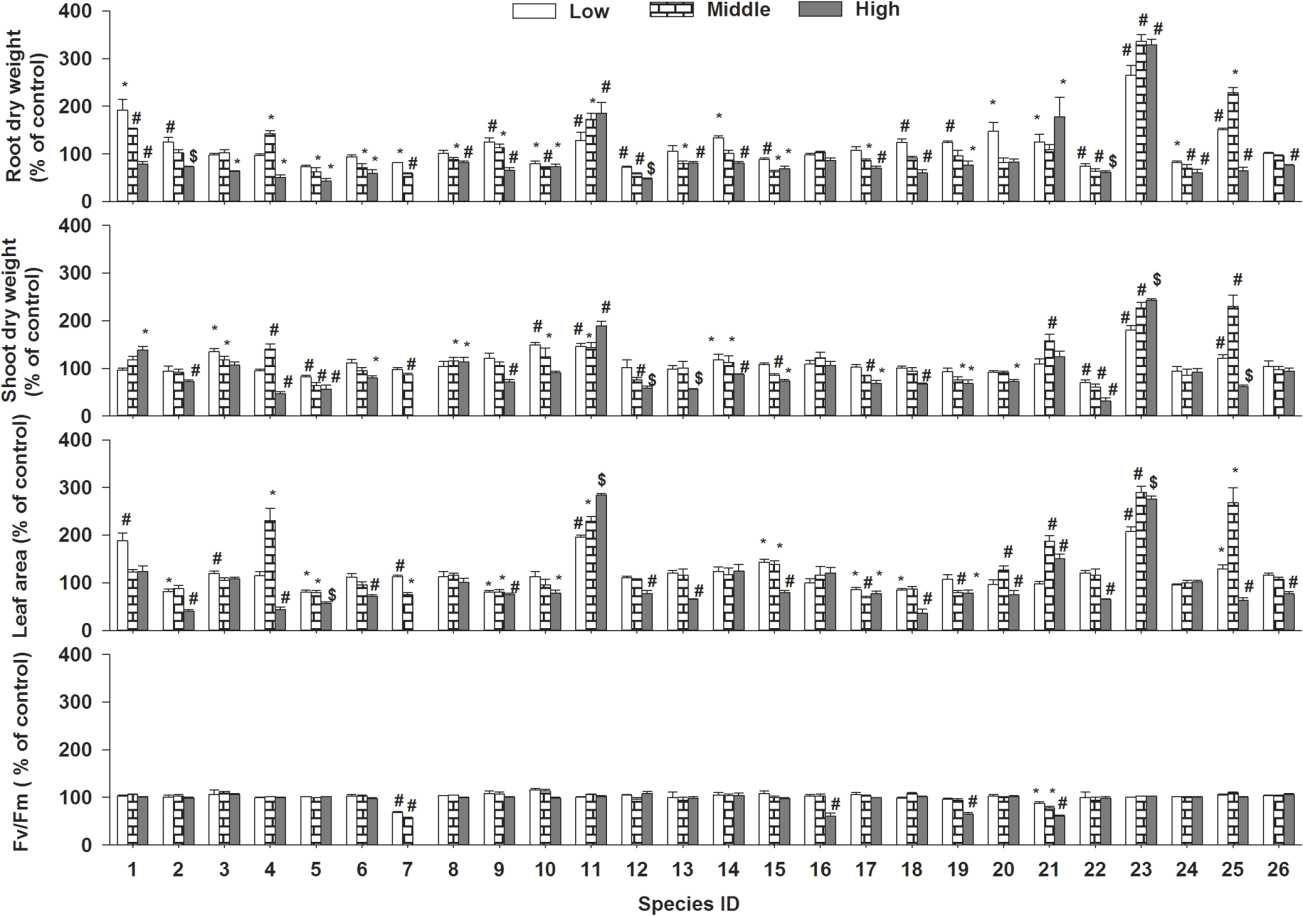
The allelopathic effects of Mikania on seedlings root dry weight, shoot dry weight, leaf areaand Fv/Fm of the 26 target species at three concentrations; bars represent standard errors;“*”: p<0.05; “#”: p<0.01; “$”: p<0.001.

Similar to effects on seed germination, the allelopathic effects on seedling root dry weight, shoot dry weight, leaf area and Fv/Fm of the 26 target species were significantly restrained and the degree of inhibition was increased with concentration, while the mean allelopathic effects of the 26 target species were not negatively significantly (Figs 2, 4 and 5, ANOVA, p<0.05, Table 2). As the effects on seed germination, the allelopathic effects on seedling growth were different among the 26 target species (Figs 2, 4 and 5, ANOVA, p<0.05, Table 2). Though the allelopathic effects on seedling growth were neutral or stimulated for some species as effects on seed germination in low and middle concentration, the percent of these effects on seedling growth was larger than that of seed germination (Figs 1, 2 and 4). Different to effects of seed germination, however, seedling root dry weight, shoot dry weight and leaf areas of three species (*Lolium perenne*, *Trifolium repens*,*Rumex aquaticus*) even stimulated in high concentration and lower than 50% of the target species were negatively affected and most of the species were neutrally affected (Figs 4 and 5).

### Different reactions of Legume VS non-legume and native VS exotic species

**Fig 6:**
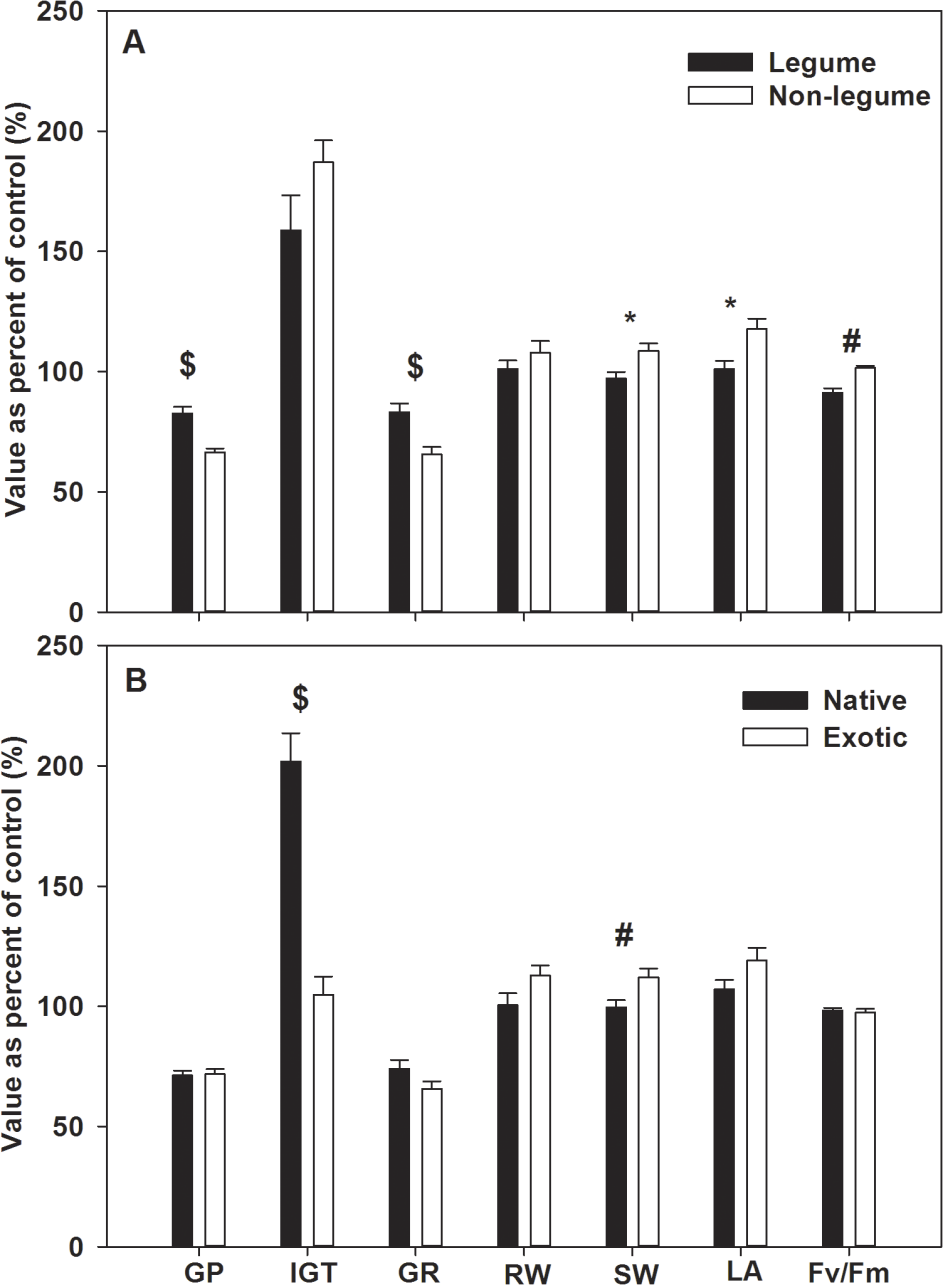
The allelopathic effects of Mikania on seed germination percent (GP), initial germination time (IGT), germination rate (GR), seedlings root dry weight (RW), shoot dry weight (SW), leaf area (LA) and Fv/Fm of the 26 target species between legume and non-legume species (A) and between native and exotic species (B); bars represent standard errors;““#”: p<0.01; “$”: p<0.001.

Compared responses of legume with those of non-legume species, germination percent and germination rate of non-legume species were stronger negatively affected than legume species (ANOVA, p<0.001), and the effects on Fv/Fm, shoot dry weight and leaf area were adverse (ANOVA, p<0.05) while effects on initial germination time and root dry weight were not significantly different (ANOVA, p>0.05) between these two groups (Fig 3). As for native and exotic plants, only the effects on initial germination time and shoot dry weight of exotic species were weaker than native species (ANOVA, p<0.01) and the effects on germination percent,germination rate, root dry weight, leaf area and Fv/Fm were not significantly different (ANOVA, p>0.05) between these two groups (Fig 6).

## Discussion

It is generally regarded that leaf has more allelochemicals than other organs [42–46], and allelochemicals are often water soluble [46], because almost all natural allelochemicals in plants’ aboveground tissues are leached into soil by water [47]; For *Mikania*, many allelochemicals from aqueous extract of leaf tissues have been successfully isolated and identified [32, 36, 48], so leaf aqueous extract (LAE) of *Mikania* was used in this experiment.

The control (distilled water) germination percentage of the 26 target species was all over than 80% (See S1 Fig), so germination of all the species was considered not disturbed by dormancy. As a common response in allelopathy [15, 21, 32, 42, 45, 49–50], seed germination and seedling growth of the target species were significantly inhibited and the inhibition degree was increased with concentration. The germination rate was a relatively more sensitive character of the target species as observed by other researchers [38, 51]. Delayed germination due to long initial germination time and slow germination rate resulted in smaller seedling and finally made it in disadvantages in the following competition for survival and growth [52–54]. While, the allelopathic effects on different measured parameters varied greatly among the target species as former researches [55–56], and the 26 species showed greatly different allelochemical-resistance to aqueous extract of *Mikania*. This agreed with other former studies [57–58], because there were different evolutionary history and varying resistance to allelochemicals among these species [21, 33, 57], this gave us an indication that some species were more allelochemical-resistant and could be screened for restoring.

Similar to other experiments, due to direct contact, first uptake and higher allelochemicals concentration around the seedling root (radicle), the allelopathiceffects on root (radicle) were stronger than those of shoot [15, 32–33, 42, 45]. It is generally thought that root (radicle) is more sensitive than seed germination (including germination percent and emergence) to allelopathy [15, 32, 42], in contrast, our results showed that seed germination characters were more negatively affected (from 15.38–46.15% in low concentration to 88.46–100% in high concentration) than seedling growth indices (from 7.69–26.92% in low concentration to 15.38–46.15% in high concentration) and even root dry weight, shoot dry weight and leaf areas of *L*. *perenne*, *T*. *repens* and*R*. *aquaticus* were promoted (Figs 4, 5 and 6). It might because that their seedlings used to measure were come from the seed affected by allelochemicals or the experiment time was so short or the seedlings were too young for their experiments and different treatment means might also partially interpret the different results [59–60]. As found by former studies, seedling growth of the three species (*L*. *perenne*, *T*. *repens* and*R*. *aquaticus*) in this experiment as some species in others could be stimulated even by the high allelochemicals or high concentration of aqueous extracts [61–62], because these species could make good use of the allelochemicals such as using them as fertilizer or have functionally adapted to the moderate concentration of allelochemicals or have the strong enough ability to detoxicate allelochemicals [62–64]. Accordingly, these three species are well potential for allelochemical-resistant species in restoring habitats invaded by *Mikania*. Furthermore, since seedling growth was less sensitive to *Mikania* extracts than seed germination and natural seedlings are more competitive than seedlings from seeds affected by allelopathic effects as mentioned above, seedlings are more suitable than seeds for restoration of *Mikania*-invaded habitats.

Seeds of legume species are larger (ANOVA, p = 0.069) and have more nutrition (energy) to invest resisting allelopathy [15, 32], so the seed germination of legume species was more weakly influenced, on the contrary, seedling growth of non-legume species was more strongly affected because allelochemicals could affect signal communication between roots of legume species and rhizobial bacteria and result in a reduction of nodulation formation and the following decrease of nitrogen fixation in legume species [24–26], which affected the nitrogen availability of legume species rather than non-legume species. In general, native plants were more strongly affected by *Mikania* allelochemicals than exotic plants, it was possible that some exotic plants from the same region with *Mikania* were more allelopathic-resistant to allelochemicals [65]. So, restoring with naturalized exotic species, which are from the same region with the invasive plant, in the invaded habitats would be easier. Therefore, using naturalized exotic non-legume species to restore *Mikania*-invaded habitats is more applicable than using native legume species.

## Conclusion

Seed germination was more strongly negatively affected by LAE of *Mikania* than seedling growth. Responses of seed germination and seed growth to LAE of *Mikania* differed differently among the target species. LAE of *Mikania* more strongly negatively affected seed germination, but less strongly negatively affected seedling growth, in non-legume species than in legume species. LAE of *Mikania* more strongly negatively affected seed germination and seedling growth in native species than naturalized exotic species. Therefore, naturalized exotic non-legume seedlings are more suitable than seeds of native legume species for restoration of *Mikania*-invaded habitats.

## Supporting Information

S1 Fig Germination percent of the 26 study species with distilled water control. (TIF)
