# Supplementary figures and images for: Screening Allelochemical-Resistant Species of the Alien Invasive Mikania micrantha for Restoration in South China
Source: PLoS One. 2015 Jul 15;10(7):e0132967. doi: 10.1371/journal.pone.0132967 (PMC4503634; doi:10.1371/journal.pone.0132967)

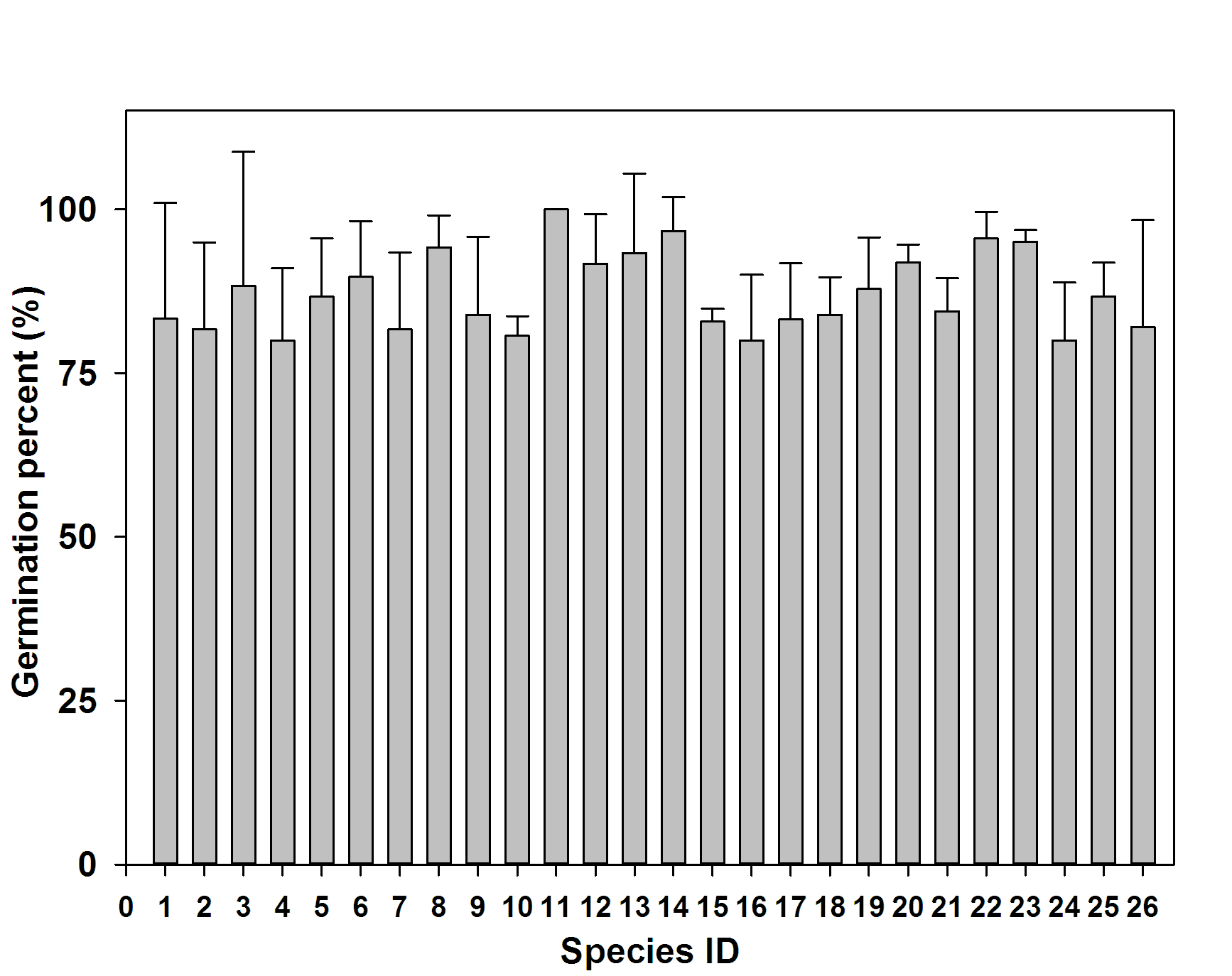

Supplement: S1 Fig — (TIF) [file pone.0132967.s001.TIF]
